# Evaluation of school absenteeism data for early outbreak detection, New York City

**DOI:** 10.1186/1471-2458-5-105

**Published:** 2005-10-07

**Authors:** Melanie Besculides, Richard Heffernan, Farzad Mostashari, Don Weiss

**Affiliations:** 1 Communicable Disease, New York City Department of Health and Mental Hygiene, New York, NY, USA; 2 Mathematica Policy Research, Inc, Cambridge, MA, USA; 3 Epidemiology and Surveillance, New York City Department of Health and Mental Hygiene, New York, NY, USA

## Abstract

**Background:**

School absenteeism data may have utility as an early indicator of disease outbreaks, however their value should be critically examined. This paper describes an evaluation of the utility of school absenteeism data for early outbreak detection in New York City (NYC).

**Methods:**

To assess citywide temporal trends in absenteeism, we downloaded three years (2001–02, 2002–03, 2003–04) of daily school attendance data from the NYC Department of Education (DOE) website. We applied the CuSum method to identify aberrations in the adjusted daily percent absent. A spatial scan statistic was used to assess geographic clustering in absenteeism for the 2001–02 academic year.

**Results:**

Moderate increases in absenteeism were observed among children during peak influenza season. Spatial analysis detected 790 significant clusters of absenteeism among elementary school children (p < 0.01), two of which occurred during a previously reported outbreak.

**Conclusion:**

Monitoring school absenteeism may be moderately useful for detecting large citywide epidemics, however, school-level data were noisy and we were unable to demonstrate any practical value in using cluster analysis to detect localized outbreaks. Based on these results, we will not implement prospective monitoring of school absenteeism data, but are evaluating the utility of more specific school-based data for outbreak detection.

## Background

Public health agencies nationwide are developing surveillance systems using non-traditional data sources to enhance outbreak detection capacity. The New York City Department of Health and Mental Hygiene (DOHMH), for example, conducts daily monitoring of ambulance dispatch calls, emergency department (ED) visits, pharmacy sales and worker absenteeism [1-3]. Each of these "syndromic" surveillance systems relies on non-specific, pre-diagnostic data. Having multiple, complementary systems may increase or decrease detection performance. Findings from one data stream can support or contradict findings from another, providing additional evidence on which to decide whether a syndromic signal requires a public health response.

One data source that has been considered for use in syndromic surveillance is school absenteeism records. Reports of elevated school absenteeism made to the Milwaukee Department of Health during the 1993 *Cryptosporidium *outbreak preceded the recognition of the outbreak [4]. Monitoring of school absenteeism has also been incorporated into the Electronic Surveillance System for the Early Notification of Community-based Epidemics (ESSENCE II) program [5].

Prior to committing public health resources to establishing and maintaining a new syndromic surveillance system, however, it is important to evaluate its potential usefulness. Resources are limited, and even simple systems require staff time and equipment to process data, conduct the analyses and respond to signals. Using a recently developed framework for evaluating early outbreak detection systems as a guide [6], this paper evaluates whether daily monitoring of school absenteeism data is useful for early outbreak detection in New York City considering the use of other syndromic systems. We use three influenza seasons to test the utility of the data. The seasons varied in severity, with 2001–02 characterized as severe, 2002–03 as mild, and 2003–04 as moderate.

## Methods

There are approximately 1.1 million students enrolled in the New York City (NYC) public school system. At each of the 1,160 schools, absences are recorded on a "bubble sheet" during a designated homeroom class period. Bubble sheets are forwarded to the school's administrative offices and scanned into a local database that automatically transmits the data to a central database at the NYC Department of Education (DOE). Preliminary data are available by noon each day, however these are subject to correction over the next few days for students who, for example, arrive late. Reason for absence is not recorded.

For the purposes of this evaluation, we downloaded attendance data for the 2001–02, 2002–03, and 2003–04 academic years from the DOE website [7]. Data consisted of the daily percent present (i.e., not absent) aggregated by 'Community School Districts,' which include elementary and middle school children (kindergarten through 8^th ^grade), and 'High School' (9^th ^through 12^th ^grade). We calculated the median daily absentee rates separately among elementary/middle and high school students over the three-year period, and examined whether the absentee rates differed between the two groups. We hypothesized that absenteeism would be significantly higher among the older group of students, making age group-specific analyses necessary. We also assessed whether absenteeism varied by day of week and whether it was higher on days scheduled for parent teacher conferences, state exams, or half days, so that we could control for such days. Statistical significance of differences was measured using the Wilcoxon-signed rank test.

To identify days on which prospective surveillance would have indicated a statistically significant overall increase in absenteeism – or 'signal' – and specifically to determine whether the system could provide timely indications of community-wide influenza, we analyzed daily data from October 1, 2001 through June 25, 2004 retrospectively. The analysis mimicked prospective monitoring, specifically, all data from September 15, 2001 up to and including the day of analysis were used in each days analysis, but no future data. Separate analyses were carried out for elementary/middle and high school students.

There are many reasons why students are absent from school that are unrelated to illness and we wanted to control for these reasons whenever possible. Several steps were therefore taken to minimize the number of false positive signals that would normally result from a time-series analysis on data with many extreme data points (i.e., days with increased absenteeism). We removed extreme data points with a known explanation for absenteeism (e.g., Halloween) from the analysis because they were uninformative. Next, we adjusted the observed daily percent absent using a linear regression model, based on an existing DOHMH ambulance dispatch surveillance model [1]. The percent absent was modeled as a linear function of day-of-week (parameterized as 4 dummy variables with Tuesday as reference) and whether or not the day was a scheduled low attendance day (clerical half-days, parent-teacher conference days, and state exam days). We compared daily percentages with a 14 day baseline using a modified cumulative sums (CuSum) method (8). The two modifications were: 1) We eliminated from the baseline any day on which the residual from the regression model was more than two times the standard error of all residuals. This reduced the influence of extreme, uninformative data points on the baseline mean and standard deviation. 2) We terminated CuSum signals if the percent absent returned to within 0.5 standard deviations of the baseline mean. This reduced the number of multi-day signals that were due to a spike in absenteeism on one day that was extreme enough to cause signals for two or three consecutive days. The daily percent absent was plotted along with any CuSum C1, C2 or C3 signal [8], the daily number of emergency department (ED) patients ages 5–17 complaining of fever or influenza-like illness from an existing ED surveillance system [2], and the weekly number of influenza A and B isolates identified at NYC reference laboratories.

To assess geographic clustering in absenteeism we obtained a more detailed dataset from the DOE, which consisted of the daily number of students registered and absent by school and grade during the 2001–02 academic year. Spatial clustering by school location was assessed using a modified, purely spatial scan statistic [9,10] with a 30-day baseline, 1-day maximum temporal window and 20% (of registered students) maximum spatial window. More than one significant spatial cluster per day was possible and clusters of absenteeism could occur at a single school or several schools in a contiguous geographic area. We evaluated whether this method would have detected the sole gastrointestinal school outbreak reported during the 2001–02 school year. Analyses were carried out using SAS version 8.0 (SAS Institute, Cary, NC) and SaTScan version 4.0.3 (available free at [11]).

## Results

### Extreme, uninformative data points

Many extreme uninformative data points were removed from the dataset including 42 days just prior to or after a holiday, all 39 days during the first week and last two weeks of school, 7 snow days, and Halloween. In all, 99 (18%) of 538 elementary/middle school days and 104 (19%) of 541 high school days were removed.

### Citywide analysis

**Figure 1:**
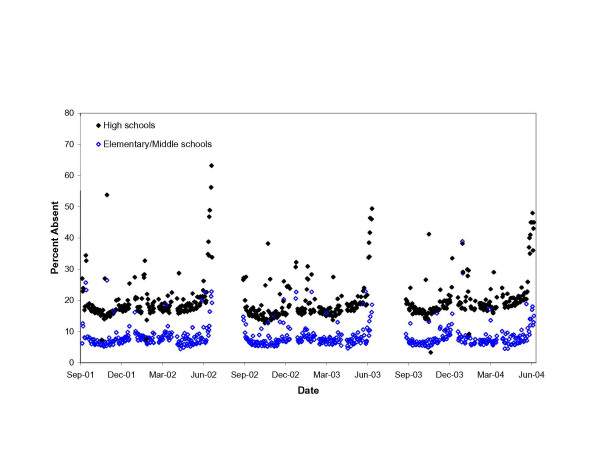
Unadjusted daily percent absent among elementary/middle school and high school students in the New York City school system, September 2001 through June 2004.

The median, unadjusted, daily percent absent over the three-year period was 7.3% (range 4.4%-38.9%) among elementary/middle school children and 17.8% (range 3.3%-63.2%) among high school students (Wilcoxon p < 0.01) (see Figure 1). Absenteeism was higher on Mondays and Fridays (median 15.8%), compared to Tuesdays (15.3%), Wednesdays (14.2%) and Thursdays (14.0%) (Wilcoxon p < 0.01). Absenteeism was also more than twice as high on days scheduled for parent-teacher conferences, New York State Regents exams, or half-days (30.4%) compared to other days (13.9%) (Wilcoxon p < 0.01).

**Figure 2:**
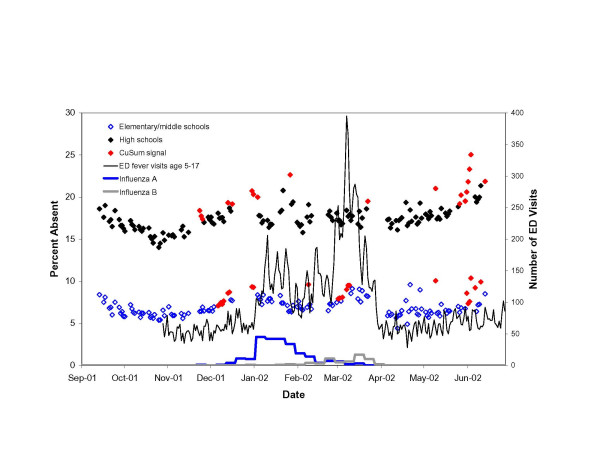
Adjusted daily percent absent among elementary/middle school and high school students in the New York City school system for the 2001–02 school year, adjusted for day-of-week and clerical days. Plotted against Emergency Department visits for fever and flu-like illness among children age 5–17 and the number of influenza A and influenza B isolates identified at three World Health Organization reference virology laboratories in New York City.

**Figure 3:**
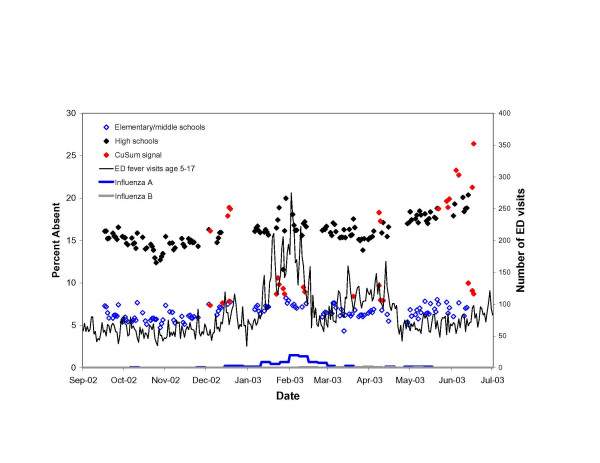
Adjusted daily percent absent among elementary/middle school and high school students in the New York City school system for the 2002–03 school year, adjusted for day-of-week and clerical days. Plotted against Emergency Department visits for fever and flu-like illness among children age 5–17 and the number of influenza A and influenza B isolates identified at three World Health Organization reference virology laboratories in New York City.

**Figure 4:**
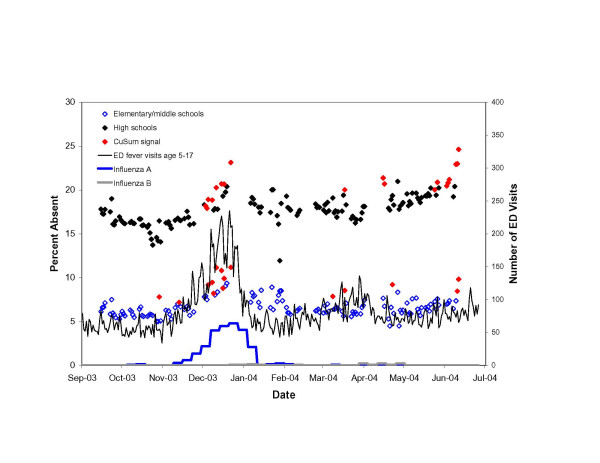
Adjusted daily percent absent among elementary/middle school and high school students in the New York City school system for the 2003–04 school year, adjusted for day-of-week and clerical days. Plotted against Emergency Department visits for fever and flu-like illness among children age 5–17 and the number of influenza A and influenza B isolates identified at three World Health Organization reference virology laboratories in New York City.

On 55 (8.0%) of 439 elementary/middle school days and on 52 (8.4%) of 437 high school days, the number of absences observed significantly exceeded the number expected (i.e., signaled) (see Figures 2, 3, 4). On many of these days, low attendance appeared to be unrelated to illness. Despite the removal of extreme, uninformative days, eleven (20%) signals among elementary/middle school children, and 24 (46%) signals among high school students occurred during late May and June as the school year came to a close. An additional 5 (9%) elementary/middle school signals and 14 (27%) high school signals occurred one day prior to or after vacation periods.

Increases in absences coincided with four community-wide outbreaks of influenza-like illness. In the first of these outbreaks, statistically significant increases in absenteeism were detected among elementary/middle school children on five days and among high school students on two days between December 10–20, 2001. This period coincided with the early stages of community-wide influenza A in New York City as evidenced by the number of influenza isolates at World Health Organization reference virology laboratories in NYC and increases in ED visits for fever/flu syndromes.

A second series of signals occurred among elementary/middle school children between March 5–13, 2002, and coincided with increases in positive influenza B isolates at reference laboratories and a season-high peak in ED visits for fever/flu and respiratory syndromes among young children. No increase in absenteeism was observed among high school students at this time (see Figure 2).

During the 2002–03 school year, a third series of signals among elementary/middle school students was observed between January 22–28, 2003. This coincided with a peak in positive influenza A isolates and ED visits for fever/flu and respiratory syndromes among children. Although sporadically elevated during this period, increases in absences among high school students were not statistically significant.

In November 2003, increases in ED visits for fever and flu-like syndrome and positive influenza A isolates at reference laboratories marked an unusually early start to community-wide influenza in New York City. School absenteeism increased exponentially during this period, with seven citywide signals among elementary/middle school students and eight among high school students from December 3–22, 2003.

### Geographic clustering analysis

Application of the spatial scan statistic identified 790 geographic areas with statistically significant clustering of absences (p ≤ 0.01) among elementary/middle school children. On average, there were three significant clusters per day with a median of five schools involved in each cluster (mean 32). The mean number of excess absences (observed – expected) in significant clusters was 287 and the mean relative risk (RR = observed/expected) was 2.1. The mean number of excess absences per school in significant clusters was 15.

Spatial cluster analysis detected an increase in absenteeism at one elementary school (school A) during a previously reported gastrointestinal outbreak. SaTScan ranks clusters from most to least significant and several schools or one school can be involved in each cluster. On two consecutive days (November 15 – 16, 2002), school A was the only school in the most significant cluster detected in the city. The number of excess absences and RR of the clusters involving school A on the 15^th ^(235 excess, 2.6 RR) and 16^th ^(152 excess, 3.6 RR) were in the top 99^th ^percentile of all clusters seen throughout the year. However, excesses in absenteeism of this magnitude were not rare; 59 schools involved in clusters had single day excesses of 200 absences or more during the year, and these signals did not stand out among the 788 other significant clusters detected during the academic year.

Among high school students during 2001–02 there were 1,018 significant (p ≤ 0.01) spatial clusters detected, a mean of four per day. The median number of schools involved in each cluster was two (mean 9, SD 18). On average there were 236 excess cases in each cluster (RR 2.1). The median excess number of students absent in each school involved in a cluster was 15 (mean 44, SD 97). There were no known localized outbreaks among high school students during the study period to test the ability of the system to detect such outbreaks.

## Discussion

School absenteeism data are collected nationwide and experience with their use in disease surveillance is likely to be of interest to many jurisdictions. Correlating peaks in daily time series from these data with positive influenza isolates is one evidenced-based approach for evaluating the ability to detect acute infectious disease outbreaks. During the 2001–02 public school year we detected moderate increases in student absenteeism associated with peak influenza A activity. Winter holiday recess likely limited continued school transmission of influenza and complicated the interpretation of signals and our ability to detect a stronger relationship. Such gaps in data limit the use of school absenteeism for tracking citywide outbreaks during these periods. We also detected a sustained increase in absenteeism among elementary/middle school students, but not high school students, during the peak of the 2001–02 influenza B season. This is consistent with the known greater susceptibility of young children to influenza B [12] and with increases in ED visits for influenza-like illness among young children but not among teenagers during this period. The relatively mild 2002–03 influenza season was associated with only a small increase in absenteeism among elementary/middle school students.

The unusually early 2003–04 influenza season afforded us the opportunity to evaluate the sensitivity of school absenteeism data to detect community-wide illness while school was in session. In this year, influenza was also severe among children because the strain of influenza circulating at the time was one that children had minimal prior cross immunity to. We observed a nearly two-fold increase in absenteeism during the peak in influenza activity. Signals generated from school absentee data, therefore, appear to require a more severe influenza epidemic among children that does not overlap with school holidays to yield a clear signal. The school absenteeism signals from the 2003–04 influenza season would have been unlikely however to have added to our existing surveillance knowledge as the ED syndromic surveillance system detected increases in influenza-like illness with earlier notification and greater specificity. The same holds true for all outbreaks identified with the school absenteeism data presented.

Spatial cluster analysis of school-level data for 2001–02 was less useful for outbreak detection. While a signal was detected during a known outbreak of gastrointestinal illness, there were several other days when similar increases were seen that appeared not to be associated with a recognized outbreak. Perhaps more importantly, routine investigations of the high number of spatial signals detected is impractical for most local or state public health agencies, given competing priorities and limited staff resources. Although some of the false positives in absenteeism at the school level could have been due to real illness, many other factors were undoubtedly involved. Given the retrospective nature of this analysis, however, we cannot confirm this, as we did not investigate the causes of signals. The magnitude of the increases detected in school absenteeism during influenza season fall far below increases detected by other syndromic surveillance systems in use by the DOHMH. Dramatic and sustained spikes in ED visits for fever and respiratory complaints and ambulance dispatch calls for fever/flu have been observed at the start of community-wide influenza outbreaks in each of the three years that this system has been in operation. Typically these influenza signals occur days to weeks before traditional influenza surveillance [1,2]. In contrast, analysis of school absenteeism data with the methods we used did not clearly differentiate between illness-related absenteeism and non-illness related absences.

## Conclusion

Monitoring school absenteeism data in New York City for outbreak detection is appealing for several reasons. Data are population-based, non-confidential, available in close to real time and can be retrieved by linking to a single central database.

Absenteeism may also provide insight into patterns of less severe illness among children who may not seek medical care. In theory, it can potentially capture illness earlier than other surveillance systems. However, in practice the data have several limitations: (1) they are non specific (i.e., the reason for absence is not known), (2) data are not collected throughout the year due to weekends, holidays and vacations, and (3) a large percentage of the days for which data are collected are uninformative, even with statistical adjustment. School absenteeism data would be more useful if they were accompanied by information about the reason students are absent.

Public health researchers should continue to seek out new sources of data to support outbreak detection, but should evaluate their usefulness before investing resources to incorporate them into routine practice. This evaluation suggests that currently available New York City school absenteeism data are too non-specific to be useful for early outbreak detection. Although it is possible that additional filtering and statistical modeling could reduce further the noise in these data, it is impractical to implement this in the public health setting and would be a poor investment given the limited information gained. Therefore, prospective surveillance of school absenteeism will not be implemented in New York City at this time. We are currently evaluating the utility of monitoring chief complaints reported by students visiting the school nurse.

## Competing interests

The author(s) declare that they have no competing interests.

## Authors' contributions

M Besculides, R Heffernan, and D Weiss conceived of the study. M Besculides and R Heffernan conducted the analysis and D Weiss and F Mostashari provided input on analysis. M Besculides led the writing and R Heffernan assisted with the writing. All authors helped interpret findings and review drafts of the manuscript.

## Pre-publication history

The pre-publication history for this paper can be accessed here:


